# Deep-sequencing identification of differentially expressed miRNAs in decidua and villus of recurrent miscarriage patients

**DOI:** 10.1007/s00404-016-4038-5

**Published:** 2016-02-15

**Authors:** Jian-mei Wang, Yan Gu, Yao Zhang, Qian Yang, Xuan Zhang, Lirong Yin, Jian Wang

**Affiliations:** The Second Hospital of Tianjin Medical University, Tianjin, China; NPFPC Key Laboratory of Contraceptive Drugs and Devices, Shanghai Institute of Planned Parenthood Research, 2140 Xie Tu Road, 200032 Shanghai, China; Institute of Biochemistry and Cell Biology, SIBS, CAS, Shanghai, China

**Keywords:** Recurrent miscarriage, miRNAs, Deep-sequencing, Villus, Decidua

## Abstract

**Purpose:**

MicroRNAs (miRNAs) are small non-coding RNA molecules that play critical roles in post-transcriptional gene expression regulation. The aim of this study was to identify differentially expressed miRNAs in decidua and villus of recurrent miscarriage (RM) patients.

**Methods:**

Participants were recruited at the outpatient Department of Gynecology and Obstetrics, The Second Hospital of Tianjin Medical University, China. Decidua and villus tissues were collected by curettage from recruited RM patients and normal pregnant women with their informed consent. MiRNAs expression profiles in decidua or villus were respectively determined by the deep-sequencing analysis. The predicated target genes of these differentially expressed miRNAs were analyzed by miRWalk. The differential expressions of four miRNAs in decidua and four miRNAs in villus between the six pairs of RM patients and normal pregnant women were confirmed by qRT-PCR analysis. The expression patterns of two predicated target genes, Bcl-2 and Pten, in the same six pairs of decidual or villus tissues were detected by Western blotting analysis, respectively.

**Results:**

Totally 18 RM patients and 15 normal pregnant women were recruited. Thirty-two miRNAs in decidua and four miRNAs in villus of RM patients were screened out to be significantly up-regulated compared to that of normal pregnant women, and five miRNAs in villus of RM patients were screened out to be remarkably down-regulated compared to that of normal pregnant women (*P* value < 0.05 and Fold change >2). These differentially expressed miRNAs were predicted to target a large number of genes that involved in cell apoptosis, p53 signaling pathway, cell cycle and other cellular bio-functions. Differential expressions of hsa-miR-516a-5p, -517a-3p, -519a-3p and -519d in decidua, as well as hsa-miR-1, -372, -100-5p and -146a-5p in villus, were validated by qRT-PCR analysis. In the decidual of RM patients, expression of hsa-miR-516a-5p, -517a-3p, -519a-3p and -519d were significantly up-regulated compared to normal pregnancy. In the villi of RM patients, expression of hsa-miR-100 and -146a-5p were significantly higher, while hsa-miR-1 and -372 were significantly lower compared to normal pregnancy. Furthermore, the expression of Bcl-2 and Pten, a predicated target gene of hsa-miR-1 or hsa-miR-372 respectively, was significantly up-regulated in the villi of RM patients.

**Conclusions:**

These data suggested that the pathogenic process of RM might be associated with the alteration of miRNAs expression profiles in decidua and villus. Especially, the aberrant placental expression of hsa-miR-1 and -372 might be involved in the progression of RM, but need to be further investigated by larger studies in the future.

## Background

Recurrent miscarriage (RM) has been defined as two or more consecutive pregnancy losses prior to the 20th week of gestation in human, and occurs in 1–2 % of pregnant women at reproductive age [1, 2], and the etiology of 68 % RM cases are unexplained [3]. Although the female causes of RM have been attributed to uterine structural defects, abnormal development of embryo, defective immunologically regulation at the maternal-fetal interface and free radical metabolism imbalance [4], the exact pathogenic mechanisms underlying RM remain unclear.

MicroRNAs (miRNAs), as a class of small non-coding RNA molecules of 21–24 nucleotides, are widely expressed in mammals to participate in post-transcriptional regulation of gene expression [5]. Mature miRNAs are incorporated into the RNA inducing silencing complex, and target-specific messenger RNAs via imperfect base pairing for translational repression or mRNA cleavage [6]. It has been estimated that miRNAs account for ~1 % of predicted genes in higher eukaryotic genomes, and 30 % of functional genes are potential targets of miRNAs [7]. Therefore, miRNAs are believed to play pivotal roles in many biological processes, including cell proliferation, differentiation and apoptosis [8]. Most miRNAs are conserved between different species, and approximately 30 % of miRNAs sequences are highly conserved between vertebrate and invertebrate genomes [9]. In particular, as they are stable and detectable in peripheral blood, miRNAs are emerging as biomarkers for clinical screening or diagnosis of human diseases [10].

In recent years, accumulating evidences indicate that abnormal expression of miRNAs is associated with multiple human reproductive disorders including endometriosis, preeclampsia, ectopic pregnancy and RM [11–13]. However, as more than 50,000 miRNAs are predicted to be presented in a mammalian cell [14, 15], and one miRNA could target multiple genes while one gene could be targeted by several miRNAs, the network of miRNAs regulation of gene expression is clearly complex and sophisticated. Therefore, it is still necessary to screen and identify specific miRNAs that are involved in the pathogenic mechanisms of RM.

Recently, the deep-sequencing analysis, a high-throughput transcriptomic approach, has been developed and successfully applied to screening differentially expressed miRNAs [16, 17]. Thus,the present study was undertaken to screen differentially expressed miRNAs in placental decidual or villi of RM patients by deep-sequencing analysis, with a view to provide new cues for the future studies on pathogenic mechanisms of RM, and to search for biomarker candidates that could be potentially used to predict adverse outcome of pregnancy at the early stage.

## Methods

### Patients and samples

All participants in this study were recruited from June 2013 to August 2013 at the outpatient department of Gynecology and Obstetrics, The Second Hospital of Tianjin Medical University, China. Trying to avoid the disturbance of confounding factors on subsequent analyses, all participants were recruited according to the same inclusion and exclusion criteria. Eighteen RM patients [age 29.61 ± 4.41 years and gestational age at sampling 8.33 ± 1.80 weeks (mean ± SD)], who had experienced at least two consecutive embryonic losses before the 12th gestational week and whose current pregnancy loss was objectively confirmed by transvaginal ultrasound exam, were recruited in the RM group. All clinical summaries about their personal history for thromboembolic disease and successful pregnancy or previous pregnancy losses were obtained. Classical risk factors such as abnormal parental karyotypes, uterine anatomical abnormalities, infectious diseases, luteal phase defects, diabetes mellitus, thyroid dysfunction and hyperprolactinemia were excluded by medical examinations. Meanwhile, 15 clinically normal pregnant women [age 29.33 ± 6.94 years and gestational age at sampling 7.33 ± 0.82 weeks (mean ± SD)], which were terminated for non-medical reasons and undergoing legal abortions around the 6–12th gestational week were recruited in the normal pregnancy group as the control. They were also checked for classical risk factor for pregnancy loss. After informed consent was obtained, decidua and villus tissues were collected by curettage from these 33 participants respectively. All the collected tissues were immediately minced into small fragments and stored in RNAlater^®^ tissue collection solution (Invitrogen, Carlsbad, CA) at −80 °C until further analyses in May 2014, namely, these tissues have been stored for 9–11 months. This study was approved by the Medical Ethics Committees of Shanghai Institute of Planned Parenthood Research (Ref # 2013-7, 2013-12). Written informed consents were obtained from all patients who provided tissue samples, and we have also obtained consents to publish research data derived from these collected samples.

### Small RNA deep-sequencing and data analyses

The total RNA used for deep sequencing was extracted from decidua and villus tissues using TRIzol reagent according to the manufacturer’s protocol (Invitrogen), respectively. The concentration of the total RNA product was measured by NanoDrop (Thermo Scientific, Wilmington, DE, USA), and the RNA integrity was checked on Bioanalyzer2100 (Agilent, Santa Clara, CA). Two hundred nanograms (200 ng) total RNA product of each case was used for preparing small RNA libraries according to the manufacturer’s instructions (Illumina, San Diego, CA), and RNase inhibitor (Invitrogen) was contained in the reverse transcription system. The cDNA libraries were sequenced on Illumina HiSeq 2000 instrument with 50-base pair single reads. Raw sequencing data was mapped to human miRNAs database (miRBase v21) using Bowtie2. Then, a Mann–Whitney test was performed to discover differentially expressed miRNAs between RM group and normal pregnancy group. These miRNAs with *P* value <0.05 and Fold change >2 were judged being significantly differentially expressed in the decidua or villus of RM patients compared to normal pregnant women. The predicted target mRNAs of these differentially expressed miRNAs were predicted by miRWalk (http://www.umm.uni-heidelbergde/apps/zmf/mirwalk/mirnatargetpub.html).

### Quantitative RT-PCR

Total RNA samples of decidua and villus tissues were extracted using TRIzol according to the manufacturer’s instructions (Invitrogen) respectively, and quantified using the NanoDrop ND-1000 (Thermo Scientific). Single-stranded cDNA was synthesized using a reverse transcription kit system (Applied Biosystems, Foster City, CA, USA). Real-time PCR was carried out using FastStart Universal SYBR Green Master (Roche Diagnostics, Welwyn Garden City, UK) and analysed using an ABI 7900 HT (Applied Biosystems). All miRNAs Assay primers used in this study were purchased commercially (RiboBio, Guangzhou, Guangdong Province, China). Primer efficiencies were determined by standard curve. Relative miRNAs expression was calculated by efficiency-corrected ΔΔCt method, normalized to the endogenous control U6 snRNA. Each sample in RM group and normal pregnancy group was measured in triplicate and the experiment was repeated for at least three times.

### Western blotting

The collected decidua and villus tissues were quickly frozen in liquid nitrogen, and granulated into fine powder. The tissue powder was homogenized in lysis buffer (Beyotime, China). The tissue lysate was centrifuged, and the supernatant was transferred into a new tube. Protein concentration was measured by the Bradford assay (Bio-Rad, Hercules, CA). Protein concentrations were determined using a standard Bradford assay, and 50 µg of total protein was separated on a 12 % acrylamide gel, and then transferred electrophoretically onto nitrocellulose membranes (Millipore, Darmstadt, Germany). Membranes were incubated overnight at 4 °C with specific primary antibodies against Bcl-2, Pten and β-actin (Santa Cruz, Santa Cruz, CA), followed by incubation with appropriate secondary antibody. The blot was developed using the PhosphaGLO AP Substrate kit (KPL, Gaithersburg, MD) according to the manufacturer’s protocol. Samples were subjected to Western blotting analysis, which was repeated in triplicates. To ensure accurate comparability between experiments, bands intensity was quantified by densitometry using ImageJ software (US National Institutes of Health, Maryland, USA) and normalized to internal control.

### Statistical analysis

All values are presented as mean ± SEM. Statistical comparisons among groups were analyzed by one-way ANOVA followed by Student’s *t* test using SPSS software package (version 19, SPSS Inc., Chicago, IL). A value of *P* < 0.05 was considered significant.

## Results

### Differentially expressed miRNAs in decidua and villus of RM patients

**Fig. 1:**
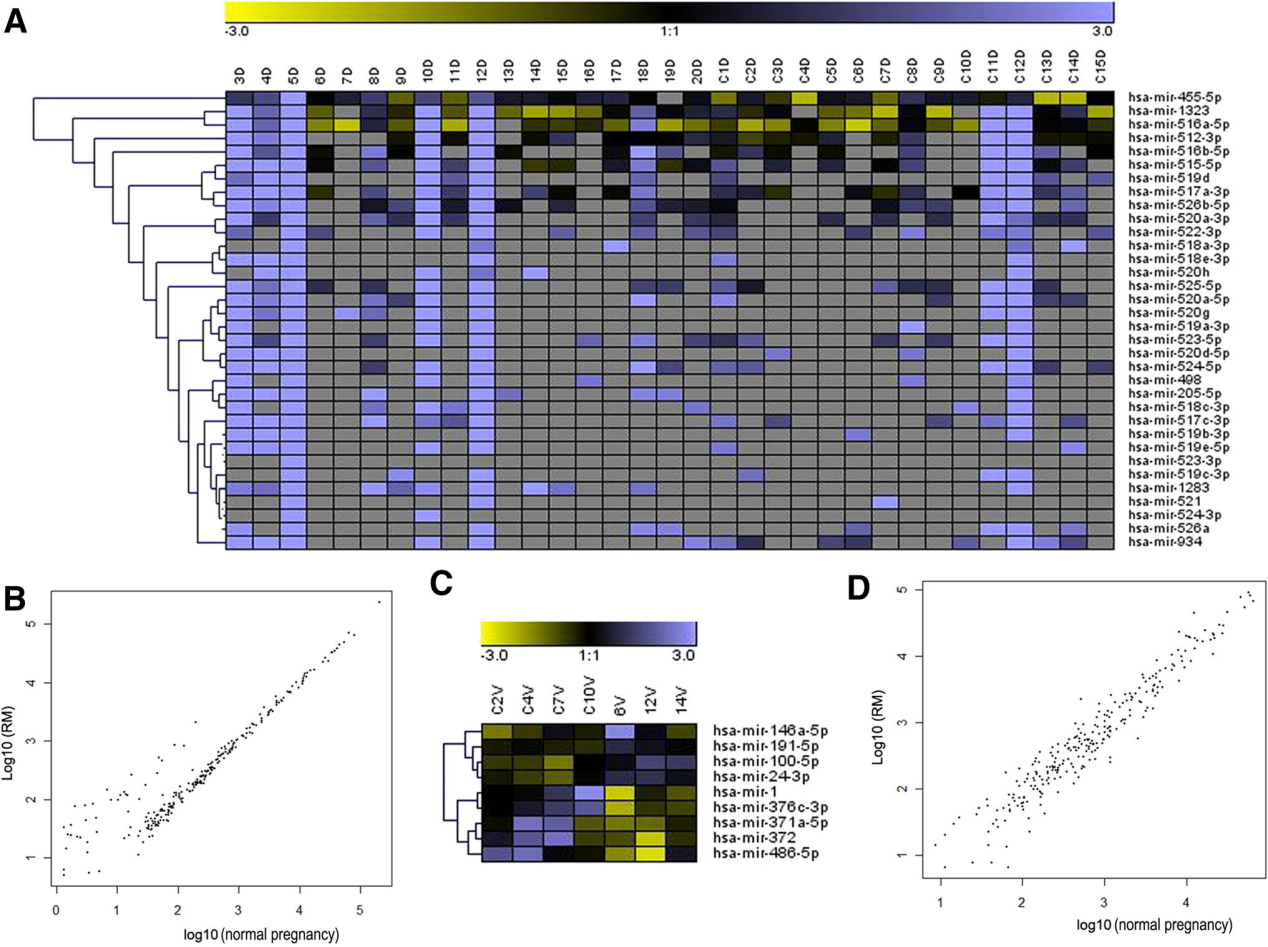
Expression profile of miRNAs in villus and decidua of RM and normal pregnancy. **a** Heatmap of differentially expressed miRNAs in decidua. **b** Log–log scatter plot comparing global miRNAs expression profiles in decidua of RM patients with those of normal pregnancy. **c** Heatmap of differentially expressed miRNAs in villus. **d** Log–log scatter plot comparing global miRNAs expression profiles in villus of RM patients with those of normal pregnancy

**Fig. 2:**
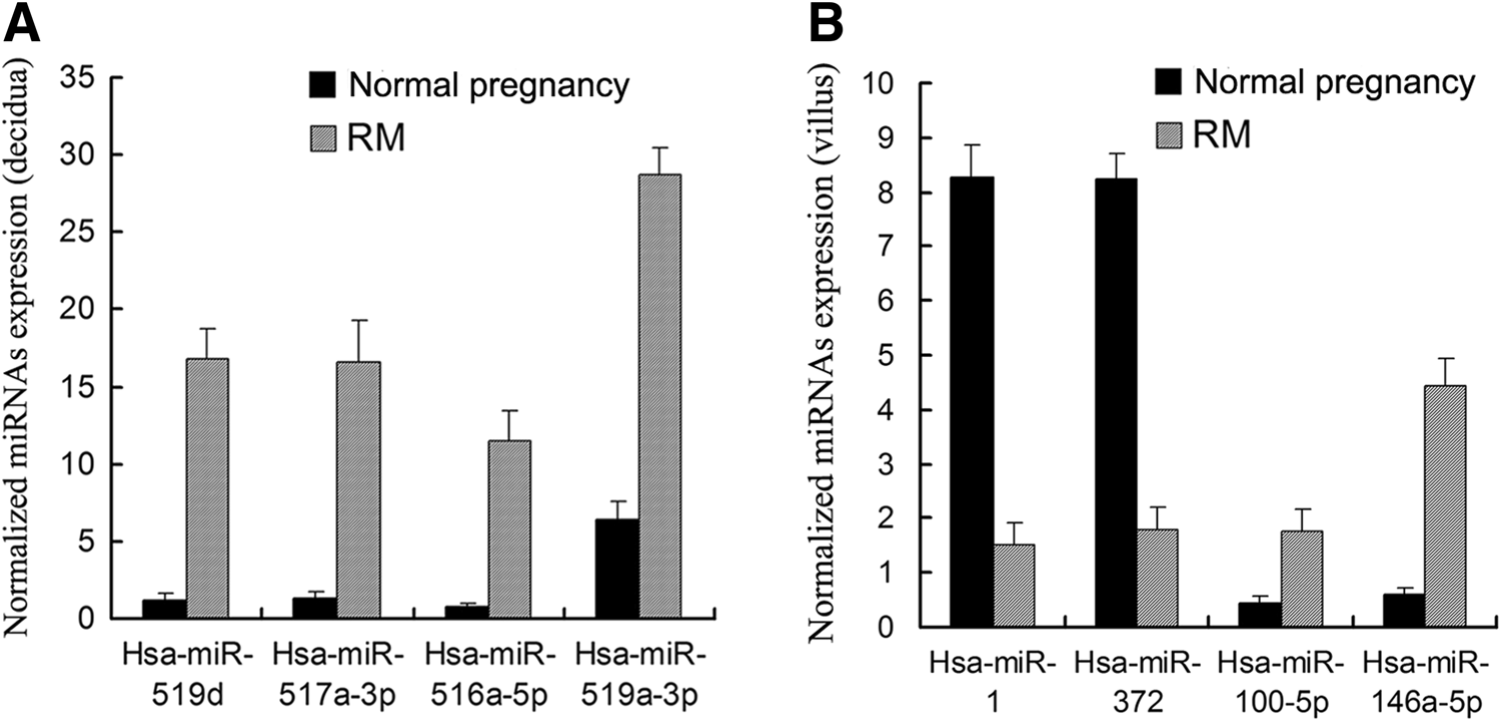
Comparison of expression levels of selected miRNAs between RM patients and normal pregnant controls. **a** Decidua. **b** Villus. *Significantly different from control (*P* < 0.05) (*n* = 6). RM recurrent miscarriage

Human decidua and villus tissues were obtained from 18 RM patients and 15 normal pregnant women. No significant differences were observed in the age and gestational period between the two groups (Table 1). Total RNA product was respectively extracted from each tissue sample. After checking the RNA integrity, all of 18 RM decidua RNA samples and 15 normal pregnancy decidua RNA samples were qualified for subsequent analysis; however, only three RM villus RNA samples (4, 10 and 12 V) and four normal pregnancy villus RNA samples (C2 V, C4 V, C7 V and C10 V) were qualified to construct cDNA libraries for deep sequencing [RNA integrities (RIN) ≥ 7 and 28 s/18 s ≥ 0.7]. Differentially expressed miRNAs were screened out by meeting our designated criteria: *P* < 0.05 and fold change >2. A total of 32 miRNAs were screened to be significantly up-regulated in decidua of RM patients, whereas a total of nine miRNAs was differentially expressed in villus of RM patients, including four up-regulated and five down-regulated miRNAs (Fig. 1; Table 2). To validate the reliability of the deep-sequencing data, we selected four miRNAs from decidua (hsa-miR-516a-5p, 517a-3p, 519a-3p and 519d) and four miRNAs from villus (hsa-miR-1, 372, 100-5p and 146a-5p) to confirm their differential expressions in decidua or villus between RM and normal pregnancy group by qRT-PCR analysis. As the villous RNA sample sizes used for deep-sequencing were too small, another three RM villous RNA samples (1, 2, 3 V,) and two normal pregnancy villous (C1 V and C8 V) RNA samples, whose integrities were qualified for qRT-PCR analysis (RIN ≥ 6 and 28 s/18 s ≥ 0.7), were also chosen to be used in qRT-PCR analysis. The qRT-PCR results showed that the expression patterns of all eight selected miRNAs were in concordance with the deep-sequencing data, indicating the deep-sequencing data were reliable (Fig. 2).

**Table 1:** Clinical characteristics of the recruited 18 RM patients and 15 normal pregnant women information about recruited 18 RM patients and 15 normal pregnancy women

Group	Sample no	Age	Gestational weeks	Childbearing history	Spontaneous abortion history
RM	1	28	8	0	4
	2	28	9	0	2
	3	35	6	0	3
	4	26	10	0	2
	5	34	12	1	2
	6	27	6	1	3
	7	30	8	0	2
	8	28	7	0	3
	9	32	8	0	2
	10	33	8	1	3
	11	42	8	1	2
	12	25	6	0	3
	13	29	8	0	2
	14	27	12	0	2
	15	28	8	0	3
	16	31	11	0	3
	17	27	7	0	4
	18	23	8	0	2
			Mean ± SD		P*
	Age		29.61 ± 4.41		>0.05
	Gestational weeks		8.33 ± 1.80		>0.05

Group	Sample no	Age	Gestational weeks	Childbearing history	Medical abortion history
Normal pregnancy	C1	31	7	1	2
	C2	42	7	1	3
	C3	26	8	0	1
	C4	40	9	1	2
	C5	31	6	1	2
	C6	29	7	0	1
	C7	27	7	1	2
	C8	26	6	1	2
	C9	19	7	0	2
	C10	37	7	1	3
	C11	37	8	1	3
	C12	22	8	0	3
	C13	27	7	0	1
	C14	24	8	0	1
	C15	22	8	0	1
			Mean ± SD		
	Age		29.33 ± 6.94		
	Gestational weeks		7.33 ± 0.82		

* Compared to that of normal pregnancy

**Table 2:** List of 41 miRNAs found by deep-sequencing analysis to be differentially expressed between RM and normal pregnancy

miRNA	Fold change (vs control)	*q* value	Style
Differentially expressed miRNAs in decidua			
hsa-miR-455-5p	2.19237434	0.04210158	Up
hsa-miR-934	4.18316511	0.00336399	Up
hsa-miR-525-5p	4.52186441	0.00003267	Up
hsa-miR-526b-5p	6.03780951	0.00000000	Up
hsa-miR-518c-3p	6.16948470	0.01993339	Up
hsa-miR-512-3p	6.17784129	0.00000000	Up
hsa-miR-1323	6.73950523	0.00000000	Up
hsa-miR-519d	8.10401668	0.00000000	Up
hsa-miR-516b-5p	8.66095224	0.00000000	Up
hsa-miR-520a-5p	9.03341830	0.00000000	Up
hsa-miR-205-5p	9.09559597	0.01232602	Up
hsa-miR-517c-3p	9.71527515	0.00000000	Up
hsa-miR-515-5p	9.83027638	0.00000000	Up
hsa-miR-523-5p	10.41084563	0.00000000	Up
hsa-miR-519b-3p	10.62396302	0.00537841	Up
hsa-miR-520a-3p	10.75501821	0.00000000	Up
hsa-miR-517a-3p	10.98821684	0.00000000	Up
hsa-miR-516a-5p	11.01941342	0.00000000	Up
hsa-miR-526a	11.10143967	0.00001016	Up
hsa-miR-520 g	12.65580255	0.00000000	Up
hsa-miR-498	14.76914649	0.00110414	Up
hsa-miR-519c-3p	14.91286942	0.00000420	Up
hsa-miR-520 h	15.48282744	0.01447064	Up
hsa-miR-524-5p	15.85564708	0.00000000	Up
hsa-miR-519e-5p	16.65645394	0.00050412	Up
hsa-miR-518e-3p	21.17422809	0.00000429	Up
hsa-miR-520d-5p	21.76428230	0.00000000	Up
hsa-miR-522-3p	21.79447724	0.00000000	Up
hsa-miR-518a-3p	25.10813623	0.00000461	Up
hsa-miR-519a-3p	27.41347031	0.00000000	Up
hsa-miR-521	28.85390185	0.00014809	Up
hsa-miR-1283	39.06915307	0.00000000	Up
Differentially expressed miRNAs in villi			
hsa-miR-1	0.21733720	0.00088976	Down
hsa-miR-372	0.22803727	0.00205597	Down
hsa-miR-371a-5p	0.25742330	0.00304584	Down
hsa-miR-376c-3p	0.28742144	0.02509974	Down
hsa-miR-486-5p	0.30897628	0.03455981	Down
hsa-miR-191-5p	2.11589617	0.00726491	Up
hsa-miR-24-3p	2.89758058	0.00004947	Up
hsa-miR-100-5p	3.70431902	0.00000000	Up
hsa-miR-146a-5p	4.45436783	0.00088976	Up

### Functional analysis of differentially expressed miRNAs in RM patients

**Fig. 3:**
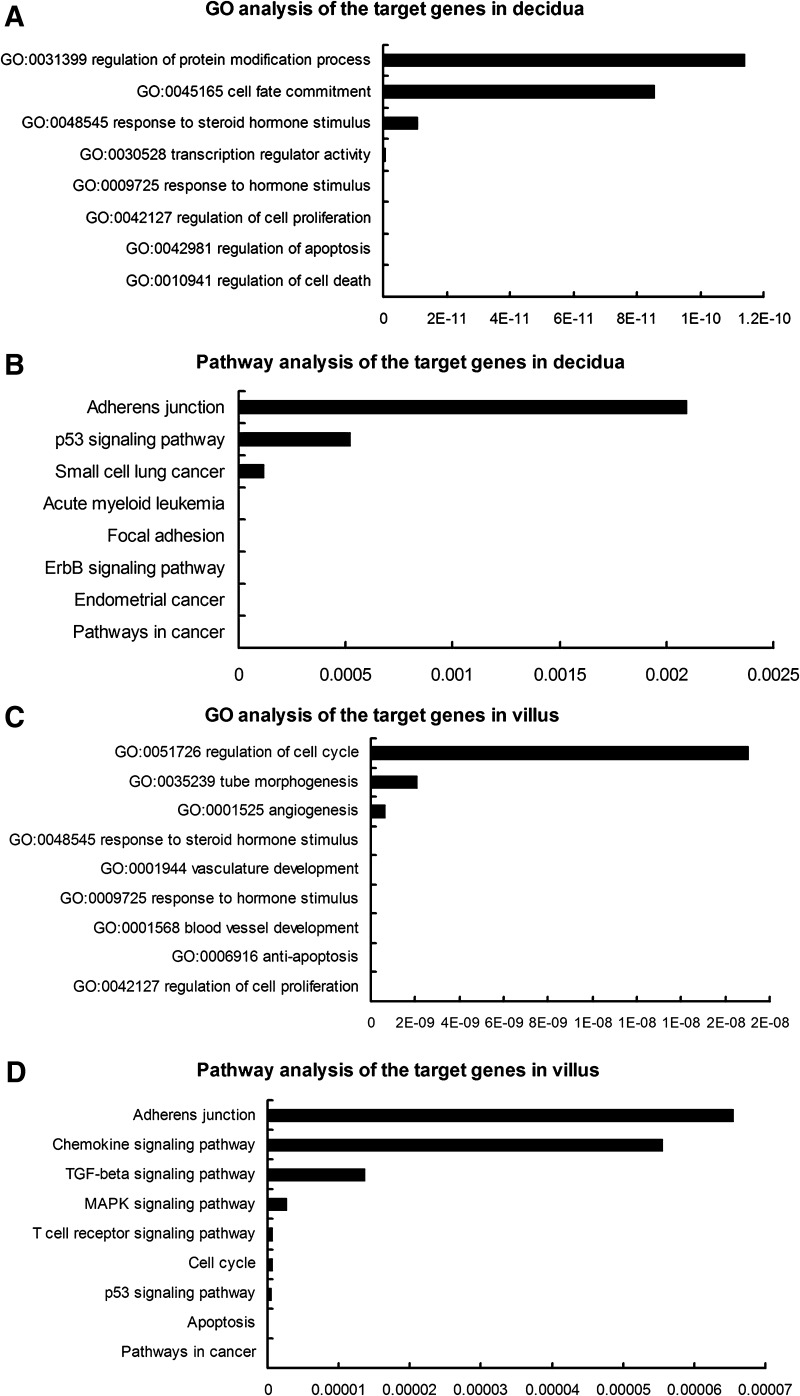
Functional analyses of differentially expressed miRNA-predicted targets in decidua and villus. **a**/**c** Results of the GO analysis of the target genes predicted by differentially expressed miRNAs in deciduas (**a**) and villus (**c**). **b**/**d** Main biological processes of genes targeted by differentially expressed miRNAs in deciduas (**b**) and villus (**d**)

A total of 252 putative target mRNAs of 32 differentially expressed miRNAs in decidua, as well as 1281 putative target mRNAs of nine differentially expressed miRNAs in villus were predicted by miRWalk. The functions of predicted target genes and molecular pathways they potentially constitute were assessed using the Gene Ontology (GO) and the Kyoto Encyclopedia of Genes and Genomes (KEGG) analyses. In decidua of RM patients, the predicted targets were significantly enriched for cell death, apoptosis, cell proliferation and response to hormone stimulus (Fig. 3a), which known to be participated in decidual development during embryo implantation. The pathway analysis showed that the predicted targets gene were involved in cancer, ErbB signaling pathway, focal adhesion and p53 signaling pathway, etc. (Fig. 3b). In the villus of RM patients, the predicted targets were significantly enriched for several biological processes known to be involved in embryonic or fetal development, such as cell proliferation, anti-apoptosis, blood vessel development (Fig. 3c). The pathway analysis showed that the predicted genes in villus were participated in apoptosis, p53-signaling pathway, cell cycle, et al. (Fig. 3d).

### Differential expressions of Bcl-2 and Pten proteins in villus of RM patients

**Fig. 4:**
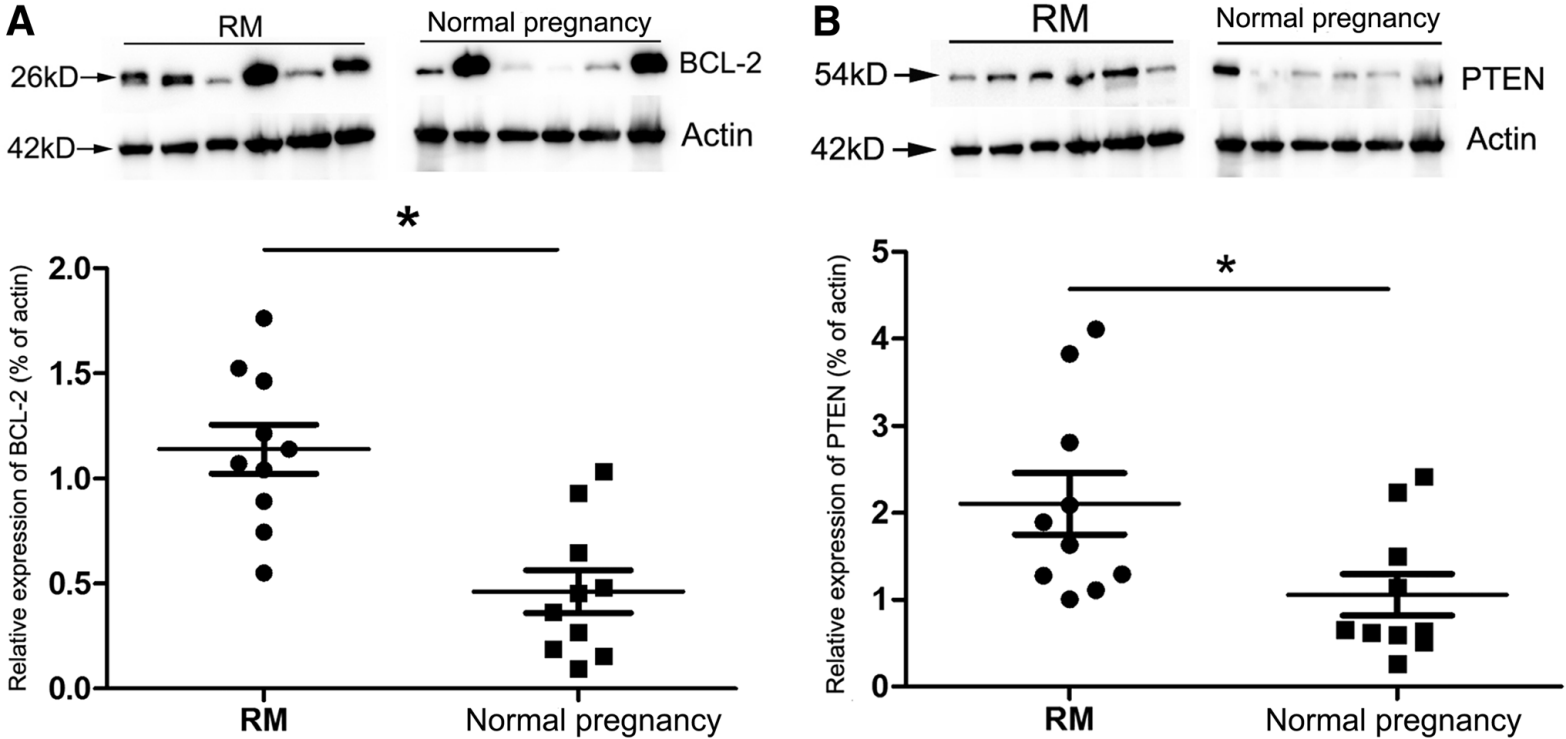
Western blot analysis of Bcl-2 and Pten expression in villus of RM and normal pregnancy. *Upper panel* representative immunoblot of three independent experiments. *Lower panel* quantification of protein levels normalized to actin. Data are the mean ± SEM of three experiments. * Significantly different (*P* < 0.05) (*n* = 6)

Given *Bcl*-*2* mRNA was predicted as a target of miR-1, and *Pten* mRNA was the predicted target of miR-372, the expression levels of Bcl-2 and Pten proteins in villus of RM patients and normal pregnant women were measured by Western blot analysis. The results showed that, the expression levels of Bcl-2 and Pten in villus of RM patients were significantly increased compared to normal pregnancy women (*P* < 0.05) (Fig. 4), in consistent with the down-regulated expressions of miR-1 and -372 in villus of RM patients.

## Discussion

In this study, we observed the differentially miRNAs expression profiles in villus and decidua of RM patients compared to that of normal pregnant women by using deep sequencing analysis. A total of 32 miRNAs were screened out to be significantly up-regualted in decidua of RM patients, while nine miRNAs were identified to be differentially expressed in placental villi of RM patients, including four up-regulated (hsa-miR-191-5p, -24-3p, -100-5p and -146a-5p) and five down-regulated (hsa-miR-1, -372, -371a-5p, -376c-3p and -486-5p) miRNAs, compared to that of normal pregnant women. We further confirmed by qRT-PCR the up-regulation of hsa-miR-516-5p, -517a-3p, -519a-3p and -519d in decidua of RM patients, and hsa-miR-100-5p and -146a-5p in villus of RM patients; as well as the down-regulation of hsa-miR-1 and -372 in villus of RM patients. Furthermore, the increased expressions of Bcl-2, a predicted target of miR-1, and Pten, a predicted target of miR-372 were observed in villus of RM patients.

Recurrent miscarriage is presently difficult to be prevented and treated for the lack of knowledge on molecular mechanisms of this disease. Thus, in order to identify novel potential targets for the clinical diagnosis or treatment of RM, we tried to establish the miRNAs expression profiles in the decidua and villus from RM patients in this study. Although we have successfully recruited 18 RM patients and 15 normal pregnant women, and all of the 33 total RNA samples from decidual tissues were qualified for deep-sequencing analysis; however, unfortunately, only three samples of villus total RNA in RM group and four samples of villus total RNA in normal pregnancy group met the requests on RNA integrity for construction of cDNA libraries. We thought that this might be resulted from a relatively long storage time of villous tissue samples (more than 9 months), and it seems that, under the same storage conditions, human decidua tissue samples should be much more stable than villus tissue samples. The size of villus samples was so small that we wonder whether or not the results of the deep-sequencing analysis were reliable. Six pairs of decidua and villus from RM patients and normal pregnancy controls were used for validation of eight selected miRNAs by qRT-PCR, and the results of qPCR were extremely consistent with deep sequencing data, indicating that the deep sequencing data were reliable.

Since each miRNA has been predicted to have a broad range of target mRNAs based on the degree of sequence homology, these 32 miRNAs in decidua and 9 miRNAs in villus could undoubtedly be involved in different cellular functions, and we wonder whether or not these cellular functions would be important for establishment and maintenance of pregnancy. The GO ananlysis provides a comprehensive source for functional genomics, and is an effective bioinformatics research tool to unify the representation of gene and gene product, and creates evidence-supported annotations to describe the biological roles of individual genomic products (e.g. genes, proteins, ncRNAs, complexes) [18]. Thus, we carried out the GO analysis for the 1533 predicted target genes (252 in decidua and 1281 in villus) of these differentially expressed miRNAs. It was revealed that, in decidua, these predicted target genes are mainly participated in cell death, apoptosis, cell proliferation and hormone stimulus, and the major KEGG pathways were cancer, ErbB signaling, focal adhesion, p53-signaling and apoptosis. Meanwhile, in villus, these target genes are mostly involved in the regulation of cell proliferation, apoptosis, blood vessel development and angiogenesis; and the major KEGG pathways analysis were apoptosis, p53-signaling and cell cycle. The pathologies that lead to RM must ultimately, either directly or indirectly, affect the interaction between the maternal endometrial (decidual) and trophoblastic tissue [19]. According to the GO analysis results, the aberrant expression of these target genes in villus might affect trophoblast invasion and placentation, while aberrant expression of these target genes in decidua could adversely impact trophoblast invasion [20]. More interestingly, given the p53-signaling pathway plays critical roles in apoptosis, and an advisable apoptosis of decidual cells, trophoblast cells and decidual immune cells are thought to be essential for the establishment and maintenance of pregnancy [21, 22], we speculated that the differentially expressed miRNAs (such as hsa-miR-519a, hsa-miR-517a, hsa-miR-205, hsa-miR-1, hsa-miR-372 and has-miR-486) which target apoptosis related gene, might involved in the regulation of p53-signaling and apoptosis might participate in the pathogenesis of RM.

Bcl-2, a key regulator of cell apoptosis, was identified as a predicted target of miR-1, a down-regulated miRNA in villus of RM patients. MiR-1 is a member of the muscle-specific miR-1 family (myomiRs) that currently consists of six members [23]. It has previously been reported that, miR-1 inhibits cell proliferation but promotes cell differentiation, and is involved in tumorigenesis as a tumor suppressor. Its expression is abnormally down-regulated in several types of cancers, including lung, prostate, colorectal cancers and rhabdomyosarcoma [24]. The abnormalities of Bcl-2 function have been implicated in many diseases including cancer, neurodegenerative disorders and autoimmune diseases [25]. It was observed in this study that, the expression level of Bcl-2 protein was significantly increased in RM villus, suggesting an inhibitory effect of miR-1 on Bcl-2, and the miR-1/Bcl-2 signaling might be involved in the progression of RM. We also noticed that, two out of six bands of Bcl-2 were higher expressed in normal pregnancy than in RM, this might be resulted from the individual differences or/and the gestational week variation. As the cell proliferation and death of trophoblast are active during the early placental development [26], the heterogeneity of Bcl-2 expression might be observed in different human villus tissues, calling for the validation by a larger sample size. Meanwhile, it is confusing that, the expression level of Bcl-2 in trophoblast cells increased during placenta development [27], and a reduced expression of Bcl-2 was in association with pregnancy loss [28]. This may be due to the multiple and complex effects of differentially expressed miRNAs on the microenvironment of decidua and villus of RM patients. In order to improve the clinical management of RM patients, great efforts have been made to search for biomarkers potentially could be used to predict adverse outcome of pregnancy at the early stage, and miRNAs present promising biomarker candidates of RM because the serum concentration of miRNA could be measured in maternal peripheral blood samples [1, 29]. MiR-1 has been identified as potential diagnostic biomarker for colorectal cancer [30]. Thus, the serum concentrations of miR-1 in RM patients should be measured and compared with normal pregnant women to explore the possibility of their acting as biomarker candidate for RM.

Pten, another important factor involved in p53-signaling pathway and apoptosis, was predicted as a target gene of miR-372, which was down-regulated in villus of RM patients. MiR-372 belongs to the mir-371-372 gene cluster, which is located on chromosome 19q13.42 [31]. Although the role of miR-372 itself in reproductive regulation has not been clear, it has been reported that, the expression level of miRNA-371 was increased from first trimester trophoblast cells to term trophoblast cells [32]. Further evidence reinforced that miR-371 cluster was up-regulated in the first trimester placentas compared to the third trimester placentas, indicating that miR-371 might play critical roles in placental development [33]. Meanwhile, it has been demonstrated that miR-372 regulated the cell cycle, apoptosis, invasion and proliferation in several types of human cancers [34], thus, it would be reasonable for us to speculate that miR-372 might also be involved in placental development. To study the correlation of miR-372 and Pten, we detected the expression of Pten in villus of RM patients, and found that the expression level of Pten was increased in RM villus, suggesting an inhibitory effect of miR-372 on Pten, and the miR-372/Pten signaling might be involved in the progression of RM. Consistently, it has been reported that, the villous expression of Pten was decreased as the pregnancy advanced, and an up-regulated expression of Pten was observed in early pregnancy loss [35, 36]. It has been shown that circulating serum mir-372 could serve as testicular germ cell cancer biomarker [37]. So, miR-372 presents the another biomarker candidate for RM that is needed to be validated in larger size studies in the future.

In this study, we have screened out a number of intriguing miRNAs expression differences between RM and normal pregnancy. To further authenticate the association between these differentially expressed miRNAs and the pathologic process of RM, a case–control cohort of RM with a considerably large size (at least 100 pairs) should be established to collect the tissue and peripheral blood samples. The aberrant expressions of miRNAs might be linked to the abnormal cellular processes in RM patients, but the hypotheses about the roles of each specific miRNA in the progression of RM are needed to be further investigated. Also, pregnancy complications are notoriously hard to study in the laboratory because of the absence of appropriate models of human pregnancy. Furthermore, we found many changes in miRNAs expression potentially affecting many different processes, and it should to be note that, as the miscarried embryos were dead, necrosis or inflammation might have occurred in the dead embryonic tissues, whereas the embryonic tissue of induced abortion was fresh, therefore the differentially expressed miRNAs identified here might be the result of miscarriage but not the causes.

## Conclusions

Collectively, a number of miRNAs were identified to be differentially expressed in decidua or villus tissues of RM patients, and these miRNAs might be involved in many bio-functions including p53-signaling and cell apoptosis. The aberrant placental expression of hsa-miR-1 and -372 might be involved in the progression of RM by targeting Bcl-2 or Pten respectively. Future studies will investigate the pathogenic roles of miR-1/Bcl-2 and miR-372/Pten pathways in RM, as well as the association of serum concentrations of miR-1, -372 and other differentially expressed miRNAs with the pathogenic process of RM.

## Abbreviations

Bcl-2: b-Cell lymphoma-2
GO analysis: Gene ontology analysis
KEGG analysis: Kyoto encyclopedia of genes and genomes
Pten: Phosphatase and tensin homolog deleted on chromosome ten
RM: Recurrent miscarriage
